# The Predicted Proteomic Network Associated with the Antiarthritic Action of Qingfu Guanjieshu in Collagen-II-Induced Arthritis in Rats

**DOI:** 10.1155/2013/582493

**Published:** 2013-05-27

**Authors:** Ting Yu Wang, Hua Zhou, Yuen Fan Wong, Pui Kei Wu, Wen-Luan Wendy Hsiao, Elaine Lai-Han Leung, Liang Liu

**Affiliations:** ^1^Department of Pharmacy, Shanghai Ninth People's Hospital, Shanghai Jiao Tong University School of Medicine, 639 Zhizaoju Road, Shanghai 200011, China; ^2^Center for Cancer and Inflammation Research, School of Chinese Medicine, Hong Kong Baptist University, Kowloon Tong, Hong Kong; ^3^State Key Laboratory of Quality Research in Chinese Medicine, Macau University of Science and Technology, Avenida Wai Long, Taipa, Macau

## Abstract

Qingfu Guanjieshu (QFGJS) is an herbal preparation for treating rheumatoid arthritis (RA). Previous studies revealed that QFGJS significantly inhibited experimental arthritis and acute inflammation, accompanied by reduction of proinflammatory cytokines and elevation of anti-inflammatory cytokines. This study aims to identify the targeted proteins and predict the proteomic network associated with the drug action of QFGJS by using 2D gel and MALDI-TOF-MS/MS techniques. Thirty female Wistar rats were evenly grouped as normal and vehicle- and QFGJS-treated CIA rats. The antiarthritic effect of QFGJS was examined with a 19-day treatment course, and the knee synovial tissues of animals from each group were obtained for 2D gel and MALDI-TOF-MS/MS analysis. Results showed that QFGJS significantly ameliorated collagen II-induced arthritis when administrated at 2.8 g/kg body weight for 19 days. 2D gel image analysis revealed 89 differentially expressed proteins in the synovial tissues among the normal and vehicle- and QFGJS-treated CIA rats from over 1000 proteins of which 63 proteins were identified by MALDI-TOF-MS/MS analysis, and 32 proteins were included for classification of functions using Gene Ontology (GO) method. Finally, 14 proteins were analyzed using bioinformatics, and a predicted proteomic network related to the anti-arthritic effect of QFGJS was established, and Pgk1 plays a central role.

## 1. Introduction

Rheumatoid arthritis (RA) is a chronic inflammatory and immunological disease characterized by an invasive synovial hyperplasia that leads to cartilage and bone destruction. RA afflicts generally 0.5~1.0% of the population worldwide and commonly leads to loss of joint function and consequently reduction of life quality and expectancy [1, 2]. The etiology and pathogenesis of RA remain still uncertain, and no ideal therapeutics from conventional medicine is available for treating RA at this moment. Complementary and alternative medicine (CAM), predominantly herbal therapy, has been reported valuable in treating RA due to its effectiveness in preventing structural damage of the arthritic joints and its highly tolerance to RA patients [3, 4].

 Herbal medicine, especially the herbal formulas, has been widely used for treating RA with promising outcomes for long history in China, Japan, and other Asian countries. QFGJS was prepared from five herbs as follows: Caulis Sinomenii 12 g, Radix Aconiti Lateralis Preparata 9 g, Rhizoma Curcumae Longae 6 g, Radix Paeoniae Alba 15 g, and Cortex Moutan 9 g. Based on the amount of these five herbs in QFGJS, the percentage composition of these five herbs were calculated as 23.53%, 17.65%, 11.76%, 29.41%, and 17.65%, respectively [5]. In our previous studies on QFGJS, it was found that QFGJS administered before the induction or after the establishment of arthritis could significantly inhibit the onset and progression of the experimental CIA and adjuvant-induced arthritis (AA) in rats, showing marked decrease of the incidence and degree of clinical symptoms of arthritis, paw volume, arthritic score, and erythrocyte sedimentation rate (ESR), as well as reduction of cartilage and bone destruction revealed by radiological and histopathological analyses [5]. Further mechanistic studies showed that the anti-arthritic effect of QFGJS was in association with significant suppression of three major pro-inflammatory cytokines (TNF-*α*, IL-1*β*, and IL-6) in serum during disease progression of AA and CIA in rats [5, 6]. However, both of the protein networks related to the pathogenesis of AA and CIA models and the drug action mechanisms of QFGJS remain unknown. 

Autoimmune conditions like RA are characterized by the complexity of systematic pathologies difficult to be elucidated by the conventional biological and biochemical technologies, while the large-scale analysis of proteins expression, interactions, and functions in human clinical or arthritic mouse samples has shown great promise for unlocking many of their pathophysiological mechanisms [7]. For treatment of autoimmune conditions using herbal medicine, there must be significant complexities both in the effective chemical components and drug action mechanisms, while the proteomic analytical technology would be one of the most powerful tools to identify the targeted proteins and networks related to drug actions. Technically, two-dimensional (2D) polyacrylamide gel electrophoresis coupled with matrix-assisted laser desorption/ionization time-of-flight mass spectrum/mass spectrum (MALDI-TOF-MS/MS) has been proven as the most efficient proteomics tool, where thousands of protein spots can be visualized simultaneously, resulting in a global view of the state of a proteome. Using statistical-based bioinformatics analysis, it is capable of employing the vast proteomic data to identify specific disease-associated or drug-targeted proteins with a high level of confidence [8, 9]. Also, changes in individual proteins can be detected and quantified through comparison of the 2D gel spots patterns from different samples.

 Accordingly, in the current study we utilized proteomics and bioinformatics technologies to identify the targeted proteins and predict proteomic networks of QFGJS responsible for its anti-arthritic effect in CIA rats as CIA is the most widely adopted animal models for investigating RA pathogenesis and examining drug actions of new therapeutics [10]. Simultaneously, proteomic profiling of the major complicated proteins and of CIA rats could be identified, which provides more scientific data in understanding the pathogenesis and characteristics of CIA at the protein level. In parallel with evaluation of the anti-arthritic action of QFGJS as did before [6], the knee synovial tissues of normal and vehicle- and QFGJS-treated CIA rats were isolated for proteomic analysis using 2D gel and MALDI-TOF-MS/MS techniques. Thereafter, Western blot assay was employed to verify QFGJS-targeted proteins in the same tissue samples, while bioinformatics technology and Genes Ontology (GO) analysis were utilized for classifying the identified proteins as well as establishing the proteomic networks involving anti-arthritic action of QFGJS in CIA rats.

## 2. Materials and Methods

### 2.1. Preparation of Collagen II and QFGJS Solutions

Collagen II (CII) solution was prepared by dissolving CII in 0.05 M acetic acid at 2 mg/mL (Chondrex 20022, Redmond, WA, USA) and emulsifying with an equal volume of incomplete Freund's adjuvant (IFA) (Chondrex 7002, Redmond, WA, USA) at 4°C using a high-speed homogenizer. QFGJS was prepared by extracting the five herbs with water, alcohol, and supercritical carbon dioxide followed by drying according to our previous reported method [6]. In brief, samples of the five herbs were refined as coarse powder by pulverization. Caulis Sinomenii was extracted with water, and the water extract was spray dried to obtain Extract 1. Radix Aconiti Lateralis Preparata and Radix Paeoniae Alba were refluxed together with 80% ethanol, and the ethanol extract was spray dried to obtain Extract 2. Cortex Moutan was extracted by supercritical CO_2_ to produce Extract 3. The residue after supercritical CO_2_ extraction was then refluxed with 80% ethanol and the ethanol extract was spray-dried to obtain Extract 4. Similarly, Rizhoma Curcumae Longae was extracted by supercritical CO_2_ to produce Extract 5. The residue after supercritical CO_2_ extraction was then refluxed with 80% ethanol, and the ethanol extract was dried with the vacuum drying technique to obtain Extract 6. Finally, Extracts 1–6 were mixed thoroughly to produce QFGJS. The quality consistence of QFGJS was demonstrated by HPLC fingerprint analysis. QFGJS solution at concentration of 0.283 g/mL was prepared by dissolving the drug (batch no. 20040822, granules) in the vehicle of 0.3% (w/v) carboxymethylcellulose (CMC) freshly before use.

### 2.2. Animals

Thirty female Wistar rats (5-6 weeks old) were purchased from the Laboratory Animal Services Center, the Chinese University of Hong Kong (Hong Kong). The animals were housed five per cage in rooms maintained at 20 ± 1°C with alternating 12-h light-dark cycle. Food and water were provided ad libitum throughout the experiments. Animals were acclimated to their surroundings over 1 week to eliminate the effect of stress prior to initiation of experiments. All of the experimental protocols involving animals and their care were approved by the Committee on Use of Human & Animal Subjects in Teaching and Research of the Hong Kong Baptist University and were carried out according to the regulations of the National Institutes of Health of USA and the Department of Health of Hong Kong Special Administrative Region.

### 2.3. Induction of CIA and QFGJS Treatment

Twenty female Wistar rats were used for induction of CIA according to the method described previously [5], while ten animals were used as normal control. Briefly, rats were intradermally injected at the base of the tail with 100 *μ*L CII solution, using a glass syringe with a locking hub and a 27-G needle. On day 7 after the primary immunization, all CIA rats were given a booster injection of CII solution with same dose. For normal rats, they were intradermally given saline in the primary immunization and booster injection.

 Previously, we examined the anti-arthritic effect of QFGJS on CIA rats at dose of 1.94 and 3.89 g/kg significantly inhibit the paw swelling and reduce the pro-inflammatory cytokinesis expression [5]. Therefore, we took the average of these two doses and used 2.8 g/kg as the working dosage in this project. The CIA rats were further randomly separated and exposed to once-daily oral administration of QFGJS (2.8 g/kg body weight) or vehicle (0.3% CMC solution) from the day after the onset of arthritis to day 30 of CIA induction. The administration volume (mL) to each rat equaled to one percent of its body weight (g).

### 2.4. Evaluation of the Development of Arthritis

The rats were inspected daily for the onset of arthritis characterized by edema and/or erythema in the paws. The incidence and severity of arthritis were evaluated using an arthritic scoring system, bi-hind paw volumes, and body weight measurement every 2 consecutive days beginning on the day when arthritic signs were first visible (around day 12 of CIA induction). In the arthritic scoring system, lesions (i.e., the clinical arthritic signs) of the four paws of each rat were graded from 0 to 4 according to the extent of both edema and erythema of the periarticular tissues; 16 was the potential maximum of the combined arthritic scores per animal. The hind paw volumes were measured using a plethysmometer chamber (7140 UGO, Basile, Comerio, Italy) and expressed as the mean volume change of both hind paws of rat. Body weight of the rats was monitored with a 0.1 g precision balance (Sartorius AG, Goettingen, Germany).

### 2.5. Synovial Tissues Sample Preparation

On day 30 of the experiment, all animals were sacrificed and both hind limbs were taken from rats with sterilized scissors. The limbs were put on the ice, and the synovia were isolated from the knee joints of both hind paws of each rat by removing the skin, muscle, fatty tissues, bone, and tendons of an individual paw. The synovial tissues were immediately snap frozen in liquid nitrogen and then stored in the refrigerator at −80°C.

In aspect of sample preparation for two-dimensional gel electrophoresis, the synovial tissues of each rat were pooled and washed with cold isotonic buffer for three times and centrifuged at 12,000 g for 1 min each time, and then the tissues were frozen in liquid nitrogen and ground into fine powder in an ice bath. For protein extraction, the powder was lysed with 2D lysis buffer (8 M urea, 4 M thiourea, 4% (w/v) CHAPS, and 40 mM DTT) plus 2 *μ*L/mL Benzonase (Calbiochem, Madison, WI). The lysates were incubated on ice for 20 min with gentle shaking and then centrifuged at 25,000 g at 4°C for 30 min. The supernatants were collected, and protein concentrations were determined by 2D Quant Kit (GE Healthcare, Chalfont St. Giles, UK) according to the manufacturer's instructions. 

### 2.6. Two-Dimensional Gel Electrophoresis

Equal amount of proteins (80 *μ*g) was processed by 2D Clean-up kit (GE Healthcare, Chalfont St. Giles, UK) to remove interfering substances according to the manufacturer's instructions. The purified protein pellets were air dried and resuspended in 250 *μ*L of rehydration buffer (8 M urea, 2 M thiourea, 4% (w/v) CHAPS, 0.5% (v/v) IPG buffer pH 3–10, 1.2% (v/v) destreak reagent (GE Healthcare, Chalfont St. Giles, UK)). Then, the resuspended proteins were subjected to 2D gel electrophoresis. In the first dimension, proteins were separated by IEF with a 13 cm Immobiline DryStrip gel of pH 3–10 (GE Healthcare, Chalfont St. Giles, UK) and rehydrated in a standard strip holder (GE Healthcare, Chalfont St. Giles, UK) at 30 V for 5 hr and then at 60 V for further 5 hr. The protein samples were then separated on a 50 *μ*A/strip using the Ettan IPGphor 3 System (GE Healthcare, Chalfont St. Giles, UK) with a program of stepwise increase in voltage for 65860 Vh at 20°C using the following program: 100 V step-n-hold for 2 hrs; 500 V step-n-hold for 1 hr; 1000 V step-n-hold for 1 hr; 5000 V step-n-hold for 1 hr; 8000 V gradient for 1 hr; 8000 V step-n-hold for 50000 Vhr. After the first dimension, the strips were equilibrated in SDS equilibration buffer (6 M urea, 75 mM Tris-HCl (pH 8.8), 30% glycerol (v/v), 2% SDS (w/v), and 0.002% (w/v) bromophenol blue) containing 10 mg/mL DTT for 15 min, and thereafter in the SDS equilibration buffer containing 25 mg/mL iodoacetamide (IAA) for 15 min. After equilibration, proteins were separated on 12.5% SDS polyacrylamide gel Laemmli buffer system in an SE 600 standard vertical electrophoresis unit (GE Healthcare, Chalfont St. Giles, UK). The IPG strips were sealed in place with 1% agarose solution. The second dimension electrophoresis was performed at 30 mA at room temperature. Finally, 2D gels were silver stained using a protocol compatible with mass spectrometry for protein spot visualization [11]. The silver stained gels were then scanned with an Image Scanner III (GE Healthcare, Chalfont St. Giles, UK) and analyzed with Progenesis SameSpots software (Nonlinear Dynamics Ltd., UK).

### 2.7. In-Gel Trypsin Digestion, Mass Spectrometry Analysis, and Database Searching

In-gel trypsin digestion, MS analysis, and database searching were subsequently performed. Protein spots showing significantly altered expression levels among three groups of the synovium samples were excised in duplicate from the silver-stained gels and identified by MALDI-TOF-MS/MS. Briefly, the gel plugs were dehydrated with acetonitrile (ACN), which were then removed and then were vacuum dried. Next, 20 *μ*L of 10 mM DTT in 10 mM NH_4_HCO_3_ was added, and the proteins were reduced for 30 min at 56°C. The gel plugs were then dehydrated with 200 *μ*L ACN. The supernatants were subsequently replaced with 20 *μ*L of 55 mM iodoacetamide (IAA) in 10 mM NH_4_HCO_3_. After 20 min incubation at room temperature in the dark, IAA solution was removed. The gel plugs were washed with 50% ACN in 10 mM NH_4_HCO_3_ and dehydrated with 100% ACN followed by drying in a vacuum centrifuge. The completely dried gel plugs were incubated with trypsin (trypsin gold, mass spectrometry grade, Promega, USA) solution (12.5 *μ*g/mL in 10 mM NH_4_HCO_3_) overnight at 37°C. Peptides were extracted with 1% TFA in 80% acetonitrile and vacuum dried at 45°C for 1.5 hr and then stored at −20°C until mass spectrometry.

MALDI samples were prepared by spotting 1-2 *μ*L digested solution onto a thin layer of *α*-cyano-4-hydroxy-cinnamic acid on the 600 *μ*m AnchorChip MALDI probe (Bruker Daltonik, Germany). After reaching dryness at room temperature, the samples were analyzed on a Bruker Autoflex III MALDI TOF/TOF Mass Spectrometer (Bruker Daltonik, Germany). MALDI-MS and MS/MS data were acquired and combined through the BioTools 3.0 program to search the protein database (Swiss-Prot 57.1, 462764 sequences; 163773385 residues) using in-house Mascot software (Matrix Science, London, UK).

### 2.8. Western Blot Analysis

Proteins were extracted from knee synovial tissues of the normal, CIA, and QFGJS-treated CIA rats according to previous protocols [12]. Forty micrograms of proteins from each group were separated on 12.5% SDS gels and transferred to polyvinylidene difluoride membranes (Millipore, MA, USA). Following the transfer, the membranes were blocked overnight at 4°C using 5% skim milk in Tris-buffered saline (TBS, 20 mM, Tris, 500 mM NaCl, pH 7.5) and then incubated with primary antibodies specifically against Pgk1, Gstp1, Aldh6a1, vimentin (Santa Cruz, Santa Cruz, CA, USA), and beta-actin (Sigma, St. Louis, MO, USA) in TBS for 1 hr at room temperature. The membranes were washed three times with TBST (TBS, 0.1% Tween 20) and then incubated with 1 : 10,000 dilution of anti-rabbit or anti-mouse IgG secondary antibodies (Zymed, South San Francisco, CA, USA) conjugated to horseradish peroxidase in 2% skim milk in TBST for 1 hr at room temperature. Finally, the membranes were washed three times, and the immunoreactive proteins were detected by enhanced chemiluminescence (ECL) using hyperfilm and ECL reagent (Amersham International Plc., Buckinghamshire, UK).

### 2.9. Protein Classification and Interaction Analysis

To assess the major biological themes of the differentially regulated proteins by QFGJS in the knee synovial tissues of CIA rats, Gene Ontology (GO) analysis (http://www.geneontology.org/) was conducted. The differentially expressed proteins in the normal rats, CIA rats, and QFGJS-treated-CIA rats were classified into different groups. Data filtering was preformed based on the gene product type, data source, species, and the term association. The ontology of biological processes, molecular functions, and cellular components was further filtered. The accession item was chosen to get the term lineage, and finally, the classification of a unique protein was determined and presented in Table 1. All the proteins identified by MODI-TOF/TOF of the three different experimental animal groups were classified with this method.

After classification by GO, proteins in the same class were chosen and analyzed by the String database (http://string.embl.de/). The tool provides analysis of the functionally related proteins that might be coregulated or physically interacted under CIA condition and upon the QFGJS treatment. Consequently, a predicted network of the newly identified proteins representing their protein-protein interactions related to CIA induction and the anti-arthritic effect of QFGJS can be established. 

### 2.10. Statistical Analysis

For evaluation of the pharmacological arthritic effect of QFGJS, data were expressed as mean ± SEM. Statistical analysis was performed with one-way ANOVA followed by post hoc test with least significant difference (LSD) method. All analyses were performed with SPSS 15.0 (SPSS Inc., Chicago, USA). Values were considered as significantly different when *P* < 0.05. For 2D gel results of proteomic analysis, differential software Progenesis SameSpots (Nonlinear Dynamics) was used for identification of the up- and downregulated spots by comparing the relative abundance of each matched protein spot among the normal groups, vehicle-treated CIA, and QFGJS-treated CIA groups (*n* = 6, for each sample). It was considered as significant when the overall fold change of the protein among the three groups of the animals was more than 1.4 and analysis of variance (ANOVA) P value lower than 0.5.

## 3. Results

### 3.1. Amelioration of CIA by QFGJS

**Figure 1:**
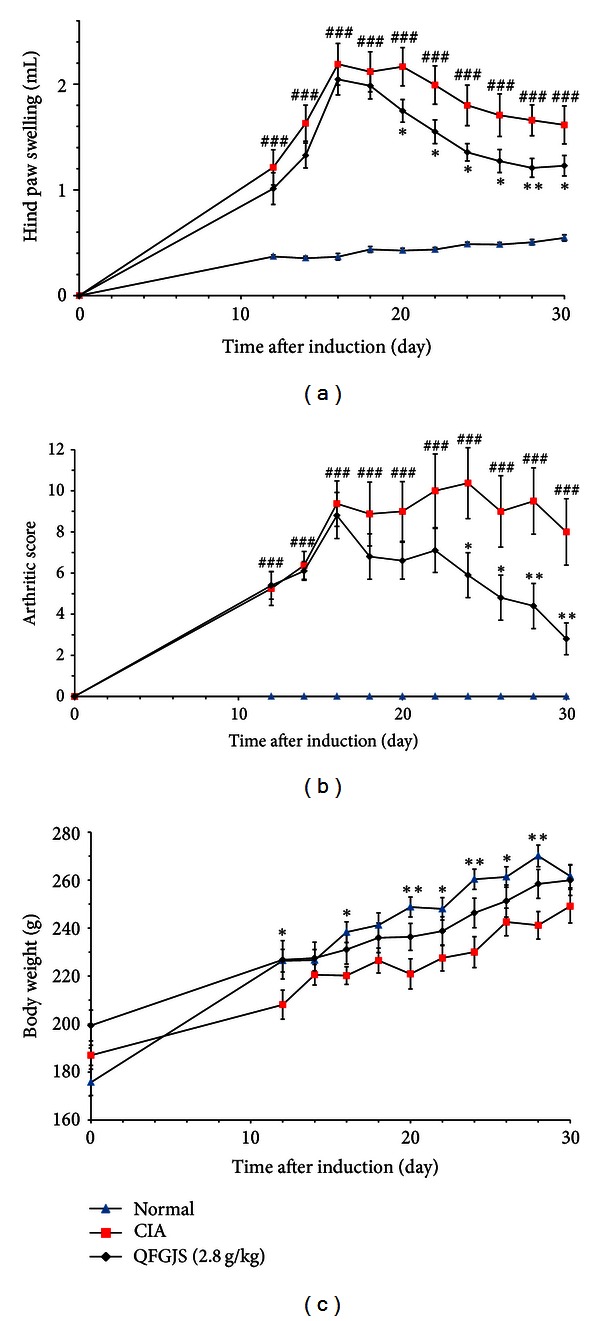
Effect of QFGJS on collagen II-induced arthritis (CIA) in rats. (a) paw swelling, (b) arthritic score, and (c) body weight. CIA was induced by intradermal injection of 100 *μ*L collagen II (CII)/incomplete Freund's adjuvant (IFA) emulsion containing 100 *μ*g of CII at the tail base of each rat, together with a booster injection of 100 *μ*g of CII in IFA on day 7 after the primary immunization. Normal rats (blue triangle) were intradermally given with saline at the primary immunization and booster injection. CIA rats were daily given with QFGJS at 2.8 g/kg body weight (black diamond) or vehicle (red square) beginning from day 12 after arthritis induction until day 30. Saline was orally given to the normal rats. Data were expressed as mean ± SEM (*n* = 10). ^###^ *P* < 0.001, normal rats versus the vehicle-treated CIA rats; *P < 0.05; **P < 0.01, QFGJS-treated CIA rats versus vehicle-treated CIA rats.

CIA was induced on day 1, while the inflammatory and arthritic lesions that appeared from day 12 onwards in CIA rats were assessed until day 30 at the end of experiment. Just from day 12, QFGJS was given once per day until day 30 as well. The results showed that QFGJS treatment markedly decreased hind paw volumes and arthritic scores from day 20 and day 24 onward, respectively (Figures 1(a) and 1(b)). Also, QFGJS demonstrated a trend against loss of body weight of CIA rats, although no statistical significance was achieved between the QFGJS-treated and control animals (Figure 1(c)). Regarding changes of the synovial tissues, the average weight of synovial tissues isolated from both knee joints of normal animals was about 10 mg, while it reached at about 15 mg in CIA rats, indicating significant cell proliferations in synovial membranes of the arthritic CIA rats. Previously, we reported the protocol of CIA in rats [5], and the experiment followed the reported protocol. Marked tissues swelling, blood vessels dilation, and accumulation of synovial fluids in the CIA rats were observed while such pathological changes were much less in the QFGJS-treated rats in comparison with the vehicle-treated CIA animals.

### 3.2. 2-D Gel Protein Spot Images of the Knee Synovial Tissues from Normal and Vehicle- and QFGJS-Treated CIA Rats

**Figure 2:**
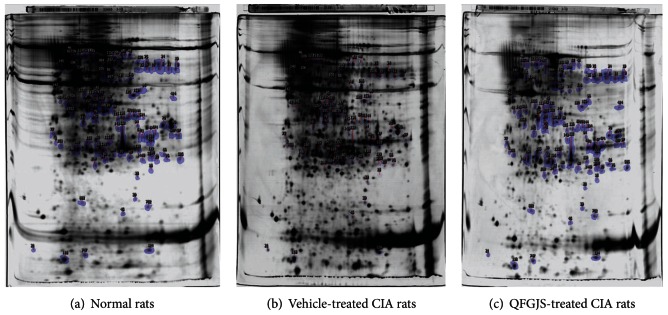
The representative images of 2D gel analysis resembling the proteome in the knee synovial tissues of the normal and vehicle- and QFGJS-treated CIA rats (*n* = 6). (a) Normal rats, (b) vehicle-treated CIA rats, and (c) QFGJS-treated CIA rats. After first dimension of IEF and second dimension of electrophoresis, the 2D gels were stained with silver solution. The gel images were scanned with Image Scanner III and analyzed with Progenesis SameSpots software. Protein spots showing the significantly altered expression levels among three groups of animals were marked and then excised for trypsin digestion followed by MALDI-TOF-MS/MS identification and database searching.

To establish a predicted potential proteomic network and identify targeted proteins of QFGJS in treating CIA rats, we employed 2D gel image technique to examine the protein expression profiling in the knee synovial tissues of the vehicle- and QFGJS-treated CIA rats, as well as normal rats. The representative 2D gel images resembling proteome of the knee synovial tissues from three groups of animals are showing in Figure 2. When we compared gels from different groups, one gel was selected as the standard, and every protein spot on this gel was distributed with a rank number by the Progenesis SameSpots software. The software has generated more than 1000 rank numbers, for example, the highest rank number in Figure 2(a) is 1349 on the upper left corner of the gel. To analyze the altered protein spots, we compiled 2D images obtained from 6 random biological replicates for each group of animals using Progenesis SameSpots software (Nonlinear Dynamics Ltd., UK). A match set was accordingly created by manual editing, and the gel with the most spots and the least background was chosen as a standard gel, and then, all of the other 17 gels were matched to this one automatically so as to match the proteins from different gels point to point. As such, all protein spots from different gels were matched to each other (Figure 2(b)). Overall, 89 differentially expressed protein spots were identified by comparing the normal- versus, vehicle- and vehicle- versus QFGJS-treated CIA rats. Such analysis on the overall fold changes of proteins expressions among three groups can ensure at the most screening of the potential proteins involved both in the pathogenesis of CIA disease and drug action mechanisms of QFGJS, so as to provide sufficient candidate proteins for further analysis using MALDI-TOF-MS/MS technique.

### 3.3. Identification of the Differentially Expressed Proteins in the Knee Synovial Tissues among Normal and Vehicle- and QFGJS-Treated CIA Rats

Among 89 differentially expressed protein spots, 63 proteins were successfully identified by MALDI-TOF-MS/MS analysis. For other 25 spots, peptide mapping could not be performed due to insufficient amount of proteins and technical limitation. Proteomic data from MS analysis were acquired and combined through the BioTools 3.0 program, and then the identities of each protein were collected after searching the protein database with Mascot software. Among 63 differentially expressed proteins, the repetitive proteins analyzed by MALDI-TOF-MS/MS (16 spots) or the unique peptides less than 2 (15 spots) analyzed by Mascot software were excluded for further classification and verification. Finally, as shown in Table 1, a total of 32 proteins have been well identified together with their Swiss-Prot accession number, theoretical molecular weight and pI, sequence coverage, Mowse scores, number of unique peptides, logarithm of average normalized volume, and overall fold changes among three groups.

 Comparing CIA rats to the normal animals, the dominant overexpression proteins in Table 1 include IgG-2a Ig gamma-2A chain C region, Gstp1 glutathione S-transferase P, Aldh6a1 methylmalonate-semialdehyde dehydrogenase, apolipoprotein C-III, isoform CRA-b, Vim vimentin, and Pgam1 phosphoglycerate mutase 1. On the other hand, both in the conditions of CIA rats and QFGJS treatment, some proteins showed a decreased expression such as Ca 3 carbonic anhydrase 3, Tpi1 triosephosphate isomerase, Anxa2 isoform short of Annexin A2, Ivd isovaleryl-CoA dehydrogenase, mitochondrial, Capzb F-actin-capping protein subunit beta, Idh3a isocitrate dehydrogenase [NAD] subunit alpha, and glyceraldehyde-3-phosphate dehydrogenase. Moreover, in animals treated with QFGJS, it was found not only to suppress proteins expressions related to the pathogenesis of arthritis but even activate expression of some proteins like Vim vimentin, C-reactive protein, and Np purine nucleoside phosphorylase. Another typical changes are phosphoglycerate kinase 1 (Pgk1) which showed only a little bit increase of protein expression under CIA condition but was significantly suppressed by QFGJS treatment, even lower than the level of normal animals. Therefore, due to such complicated changes of protein expressions under CIA condition and QFGJS treatment, our current studies should include all proteomic data from three groups of animals rather than from the treated and nontreated animals, so as to perform a more comprehensive proteomic analysis as well as achieve a global proteomic profiling upon QFGJS treatment for CIA rats. 

### 3.4. Classification of the Differentially Expressed Protein Spots among Normal and Vehicle- and QFGJS-Treated CIA Rats

Gene Ontology (GO) analysis was performed to assess the major biological themes of the differentially expressed proteins in knee synovial tissues samples among three groups of animals. As shown in Table 2, those 32 identified proteins in Table 1 could be classified into five classes upon their major known functions: 17 proteins are involved in metabolic process; 6 proteins are related to binding activity; 3 proteins can regulate biological processes; 3 proteins are in association with cellular processes, and other 3 proteins belong to the category of others.

### 3.5. Establishment of the Predicted Proteomic Network among the Major Differentially Identified Proteins Responsible for the Antiarthritic Effect of QFGJS

**Figure 3:**
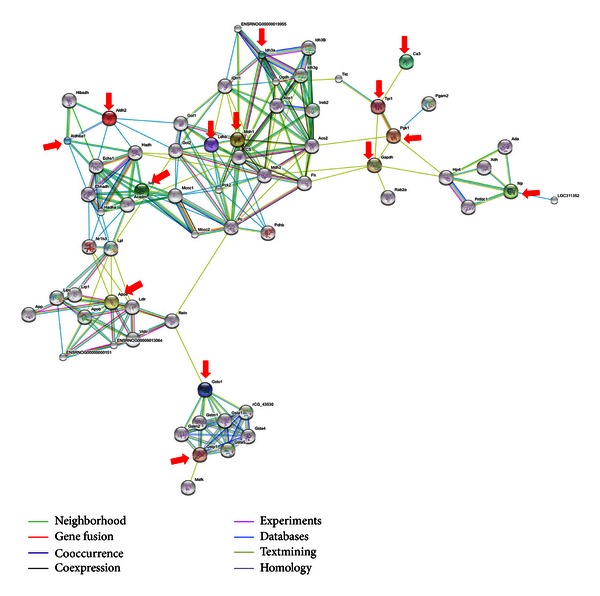
The predicted proteomic network identified by String Software resembling potential connections and interactions among the differentially expressed proteins associated with the anti-arthritic effect of QFGJS in CIA rats. The jointed lines represent the predicted protein-protein connections and correlations among the network including neighborhood, gene fusion, cooccurrence, and coexpression. The color points (arrow pointed) represent the proteins identified in the synovial tissues with functions involved in metabolic processes. The white points represent the proteins which have been reported in the literature, databases, text mining or showing homology. Although differential expression of apolipoprotein C-III, Pgam1, and Gpx1 had been demonstrated among the normal and vehicle-treated CIA and QFGJS-treated CIA rats after 2D gel analysis, these proteins with rat's origin were not found in the database of String Software; therefore, they were not included in the diagram.

After classification by GO, the majority of newly identified differentially expressed proteins in the knee synovial tissues of CIA rats responsible for the antiarthritic effect of QFGJS fell into the class of proteins which are heavily involved in metabolic process (17 proteins). Therefore, those 17 proteins were subject to further analysis using String database by which a potential proteomic network was predicted. However, as the apolipoprotein C-III, Pgam1, and Gpx1 with rat origin were not found in the database of String Software, they were excluded for building the network. Other 14 proteins (Gstp1, Tpil1, Ca 3, Ivd, Apoe, Aldh6a, Idh3a, Pgk1, Gapdh, Ldhb, Np, Mdh1, Aldh2, and Gsto1) are well connected in building of the network. As a result, predicted connections and interactions in the proteomic network between 14 proteins have been elucidated in Figure 3, which include neighborhood, gene fusion, cooccurrence, and coexpression. In this network, Pgk1 is a core protein which plays the most important role in phosphoprotein glycolysis and connects with other 13 proteins. Also, Pgk1 is the neighborhood of Tpil1 and can be translocated with Tpil1, leading to protein-protein interaction with Ca 3. Other major connections in the network are Gapdh connects Pgk1 with Np and Mdh1; Mdh1 is the neighborhood of Idh3a and Ldhb; Ldhb connects Mdh1 with Ivd, Aldh2, and Aldh6a1. Besides, Gstp1 is a homologous protein of Gsto1 and Apoe and connects them with Ivd.

### 3.6. Verification of the Targeted Proteins Responsible for the Antiarthritic Effect of QFGJS Using Western Blot Assay

**Figure 4:**
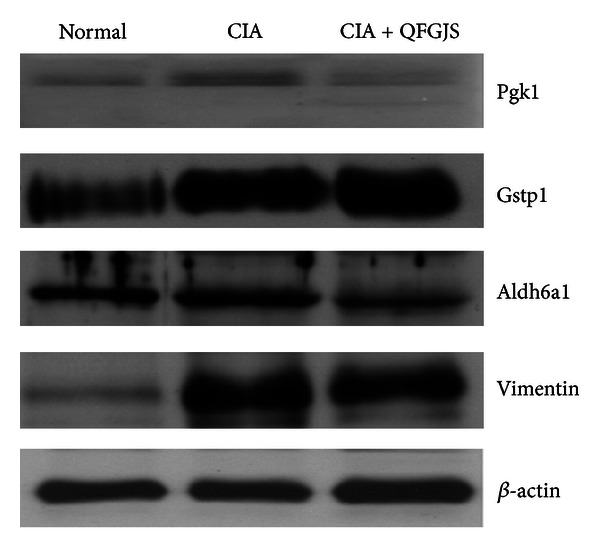
Western blot analysis for verification of the representative differentially expressed proteins. Forty micrograms of each tested protein extracted from the knee synovial tissues of normal rats, vehicle-treated CIA rats (CIA), and QFGJS-treated CIA rats (CIA + QFGJS) were separated on 12.5% SDS gels and probed with a specific primary antibody of Pgk1, Gstp1, Aldh6a1, or vimentin. Beta-actin was used as loading control and normalization. The Western blot was a representative of three individual experiments (*n* = 3).

To further verify if the major identified proteins are involved in the anti-arthritic effect of QFGJS, three proteins (Pgk1, Gstp1, and Aldh6a1), which were revealed as the most significant alteration of protein expression levels with overall fold change more than 2 and also having the most close connections and interactions in the network diagram, were selected for verification studies using Western blot assay. Vimentin was also selected for verification study because it is an important protein involving inflammatory process although QFGJS showed no suppressive effect on this protein upon 2D gel image analysis. But the Tpi1 and Car3 proteins were not employed for verification purpose due to their weak association with other proteins in the network, although their overall fold change of protein expressions was shown with more than 2. In general, the results of verification using Western blot assay demonstrated a consistent trend with alterations of the targeted proteins expression in the synovial tissue samples among the normal and vehicle- and QFGJS-treated CIA rats, compared to the results from 2D gel image analysis (Figure 4). For instance, Pgk1, Gstp1, and vimentin showed overexpression in the knee synovial tissues of vehicle-treated CIA rats, while QFGJS treatment could markedly downregulate the expression of Pgk1 protein and slightly reduce the expression of Gstp1 and vimentin. Moreover, the suppressive potency of QFGJS on Pgk1 protein expression seemed to be more prominent in the Western blot assay compared to the results using 2D gel image analysis. Interestingly, Pgk1 has been known as phosphoglycerate kinase that plays a central role of regulating signaling transduction in immunocompetent cells, while in the current study, QFGJS showed to be potent of suppressing this protein. More importantly, Pgk1 demonstrates as a core protein to be well connected with other 13 proteins in the predicted proteomic network induced by QFGJS treatment (Figure 4). Regarding expression of A1dh6a1 protein, compared to the normal animals, it demonstrated upregulatory potency in the vehicle-treated CIA rats determined by Western blot assay against its potency determined by 2D gel analysis. In QFGJS-treated CIA rats, the Aldh6a1 expression was slightly downregulated. Collectively, suppression of protein Pgk1 expression may play central roles in antiarthritis of QFGJS to CIA rats.

## 4. Discussion

The synovial membranes are a thin lining layer within joint cavities which are responsible for maintaining normal joint functions and homeostasis. The fibroblast-like synovial (FLS) cells within synovial membranes are the cells that are closely associated with the homeostatic function of joints. These cells are the primary source of articular hyaluronic acid and other glycoproteins such as lubricin [13, 14]. In chronic inflammatory and arthritic disorders such as RA, synovial membranes become the major target of a persistent inflammatory process that leads to production of thickening in the synovial lining layer, neovascularization, and lymphocyte infiltration [15–17]. Although the pathogenesis of RA remains unknown, available evidence suggests that it may involve acquisition of a combination of increased proliferative potential and resistance to apoptosis in synovial membranes. This leads to a marked increase in the number of fibroblast-like synoviocytes in synovial membranes, which participate in complex autocrine and paracrine activation networks with macrophages, lymphocytes, and dendritic cells and serve to sustain the synovitis and to enhance its destructive potential in the arthritic joints [18]. 

Several proteomic methods such as multidimensional liquid chromatography or 2-dimensional polyacrylamide gel electrophoresis (2D PAGE) in conjunction with mass spectrometry (MS) are increasingly being applied to determine differential mediators and protein markers profiling so as to identify the pathogenesis of joint diseases using samples from the synovium, cartilage, synovial fluid, and serum from patients and animals with arthritis [19–23]. Such systematic biological approaches have also been applied to explore the biomarkers and protein targets associated with therapeutic effects of drugs using RA patients' sera prior to and after therapy [24, 25]; however, application of proteomics on herbal medicine, especially on identification of the targeted proteins, remain limiting. In this study, we have applied 2D gel and MALDI-TOF-MS/MS techniques and identified the targeted proteins associated with CIA and the anti-arthritic effect of QFGJS in this rat model.

QFGJS is a pharmaceutical herbal preparation for treatment in both CIA and AA rat model; however, its molecular mechanism and target proteins have not yet been elucidated. To evaluate the influence of QFGJS on disease progression and protein expression of synovium of CIA rats, we examined the effect of QFGJS on CIA with daily treatment protocol for 19 days (from day 12 to 30 after induction of CIA) and obtained the knee joint synovial tissues for protein expression analysis. The results demonstrated that QFGJS at a dose of 2.8 g/kg body weight once daily treatment for 19 days could significantly ameliorate the arthritis induced by collagen II (Figure 1). For proteomic profiles, we have examined 18 knee synovium samples, that is, 6 random biological replicates from each group of the normal and vehicle- and QFGJS-treated CIA rats using MALDI-TOF-MS/MS technique. 2D gel analysis revealed 89 proteins differentially expressed among the normal, vehicle- and QFGJS treated CIA rats from over 1000 proteins. Of those 89 proteins, 63 proteins were identified by MALDI-TOF-MS/MS analysis (Figure 2). After elimination of the repetitive and unsatisfied proteins, 32 proteins were selected for further bioinformatics analysis (Table 1). From the data of Table 1, it indicates that simultaneously analyzing the overall fold of change of a protein involved among the normal and vehicle-treated and QFGJS-treated rats is more comprehensive than analyzing the proteomic data from the treated and nontreated animals only.

By comparing protein expression levels among three groups of animals, of those 32 proteins, 10 (IgG-2a, vimentin, Gstp1, apolipoprotein C-III, Aldh6a1, Pgam1, Gpx1, Crp, S100a4, and Apoe) were found upregulated (fold change between the normal and CIA rats: >1.4) and 8 proteins (Ca3, Tpil, Ces3, Des, Ivd, RGD1565368, Selenbp1, and Aldh2) downregulated (fold change between the normal and CIA rats: <0.71), indicating that these proteins might be involved in the pathogenesis of CIA. Of these 18 proteins, 8 upregulated proteins (IgG-2a, Gstp1, apolipoprotein C-III, Aldh6a1, Pgam1, Gpx1, S100a4, and Apoe) and 3 downregulated proteins (Ces3, Des, and Aldh2) in the QFGJS-treated animals showed a tendency of returning the normal level, suggesting that those 11 proteins might be involved in the anti-arthritic action of QFGJS. However, the expression of two proteins (vimentin and Crp) which have been upregulated in CIA were further enhanced by QFJGS treatment, while the expression of 5 proteins (Ca 3, Tpil, Ivd, RGD1565368, and Selenbp1) which have been down-regulated in CIA rats was further suppressed by QFJGS treatment. However, detailed roles of these proteins need to be further identified either in the pathogenesis of CIA or the anti-arthritic effect of CIA. Under such a situation, the Gene Ontology and proteomic network analysis were employed to elucidate the potential correlations of those 32 proteins and their roles in CIA and QFGJS treatment.

With the software of Gene Ontology, these 32 proteins were classified into five classes (Table 2): the majority of identified proteins (17 proteins) are related to metabolic processes; 6 proteins possess binding functions; 3 proteins are related to biological regulatory processes; 3 proteins are in association with cellular processes; and 3 proteins belong to other classes of proteins. And those 17 proteins involved in metabolic processes are the apolipoprotein C-III, Pgam1, Gpx1, Gstp1, Tpil1, Ca 3, Ivd, Apoe, Aldh2, Aldh6a, Idh3a, Pgk1, Gapdh, Ldhb, Np, Mdh1, and Gsto1 and were selected for predicting the proteomic network of QFGJS's action. As apolipoprotein C-III, Pgam1, and Gpx1 are not with rat origin they were excluded for further bioinformatics analysis. Subsequently, a proteomic network with predicted connections and interactions among those 14 proteins has been established in the current study (Figure 3). Especially in this proteomic network, Pgk1 was found to be a core protein with close connection to other proteins. Pgk1 is the neighborhood of and was reported to have gene fusion with Tpil1, which can interact with Ca 3. Pgk1 can also connect Gapdh with Np and Mdh1, while Mdh1 is the neighborhood of Idh3a and Ldhb. Ldhb connects Mdh1 with Ivd, Aldh2, and Aldh6a1. Gstp1 is the homologous proteins of Gstp1 and ApoE and connects with Ivd. For further verification, only proteins with overall fold change of more than 2 among three groups and involved in metabolic processes (except vimentin) were selected for further Western blot verification, of which the differential expression of Pgk1, Gstp1, Aldh6a1, and vimentin in the knee synovial samples among the normal and vehicle- and QFGJS-treated CIA rats was further confirmed by Western blot assay. Overall, the protein expression level of the core protein (Pgk1) was prominently changed in vehicle-treated CIA rats, while QFGJS treatment could restore its expression potency into an almost normal level, suggesting a strong correlation between the protein and anti-arthritic effect of QFGJS.

Moreover, Pgk1 is an important kinase for phosphoprotein glycolysis, and it was also identified as an autoantibody of RA reported by mass spectrometry analysis using 110 early untreated RA patients' sera [26]. Our data are also in line with their clinical findings, and Pgk1 was also identified as core responsive target to QFGJS in the rat CIA model, suggesting that using proteomic approach on QFGJS-treated CIA rat model is potentially useful to identify new targets with clinical relevance. Gstp1 is an enzyme that detoxifies carcinogens and protects cells against oxidative stress [27, 28], and the genetic polymorphisms of human *GSTP1* gene were reported to be associated with disease activity of RA [29]. In our current study, higher protein expressions of Gstp1 and Pgk1 in the synovial membranes of CIA rats were shown in line with the previous clinical reports, while QFGJS treatment could significantly reverse expression levels of these two proteins, indicating that QFGJS is able to reduce the autoimmunity and oxidative stress. Aldh6a1 belongs to the aldehyde dehydrogenases family of proteins and plays a role in the valine and pyrimidine catabolic pathways. The product of this protein, a mitochondrial methylmalonate semialdehyde dehydrogenase, can catalyze the irreversible oxidative decarboxylation of malonate and methylmalonate semialdehydes into acetyl- and propionyl-CoA [30, 31]. In our study, QFGJS could decrease the expression of Aldh6a1 which indicates a suppressive role of oxidative stress by QFGJS. Further studies are needed on the behavior and mechanism of interactions among those proteins in association with the anti-arthritic effect of QFGJS during metabolic process.

 Vimentin is an intermediate filament, abundantly expressed in synovial fibroblasts [32], and also a highly dynamic protein that regulates inflammatory responses assembly and disassembly via phosphorylation [33, 34]. Vimentin can be secreted by the activated macrophages through induction of TNF-*α* during inflammation, but the extracellular vimentin is essential for efficiently killing bacteria [35]. The consequence of secretion or presence of vimentin could lead to autoimmunity against these intermediate filaments. Presence of autoantibodies in autoimmune diseases such as RA has been reported in 40% RA patients' sera, and such antibodies can direct actions against the Sa antigen presence on the surface of citrullinated vimentin [36]. However, our findings in a current study showed no marked correlation between inhibition of vimentin and the antiarthritic effect of QFGJS, which needs further investigations.

 In the current study, 2D gel image and MALDI-TOF-MS/MS techniques have been successfully utilized for identification of the global proteomic profiles of the normal and vehicle- and QFGJS-treated CIA rats, as well as the targeted proteins related to the antiarthritic effect of QFGJS in CIA model, in which 32 upregulated or downregulated proteins were identified from the synovial membranes by comparing all proteomic data among three groups of animals. Bioinformatics analysis using the software of Gene Ontology classified those 32 proteins into five classes, of which 17 proteins are considered as the major corresponding ones related to the pathogenesis of CIA as well as the drug actions of QFGJS. Further analysis using String database produced a prediction of the proteomic network related to the anti-arthritic action of QFGJS, in which Pgk1 plays a central role. 

Previously, we have demonstrated five representative bioactive compounds, that is, sinomenine, paeoniflorin, paeonol, cucurmin, and hypaconitine, as the chemical markers of the pharmaceutical preparation of QFGJS [37]. Though sinomenine, paeoniflorin, paeonol, and cucurmin have an antiarthritic effect, the effects of herbal medicinal compounds contained in QFGJS on antiarthritis must not be the same with the single bioactive compounds which may be caused by multicomponents and compound-compound interactions. For example, we have previously studied the effect of pure paeonol and QFGJS containing paeonol, and the results indicated that other components in QFGJS could effectively influence the pharmacokinetic behavior and metabolic profile of paeonol in rats [38]. Therefore, combined use of all 5 herbs might probably be essential to exhibit the real treatment effect of QFGJS. However, it is interesting to further work on which components and what combination of the components providing the optimal treatment effect. Here, in the current studies we have attempted to use proteomics as the system biology platform to elucidate the network target of QFGJS, and optimization of drug combination of the network-based drug will further rely on the tighter integration of system biology and computational technologies [39].

 All in all, we have tried to link the network-based treatment principle of herbal medicine with the pharmacological target network, and we believed that the efficient use of systems biology and computational technologies for investigation of medicinal herbs and herbal preparations will function as a powerful engine for multitarget drug discovery and development of network medicine. We hoped that the QFGJS example will arouse attention on how to develop novel and better method to study the network pharmacological effect of the complex herbal formulas. 

## Figures and Tables

**Table 1:** Identification of the differentially expressed proteins in the knee synovial tissue samples of the normal and vehicle-treated and QFGJS-treated CIA rats.

Spot no.	Target protein name^a^	Swiss-Prot accession number	Theoretical Mr/pI	Sequence coverage (%)	Mowse scores	Number of unique peptides	Logarithm of average normalized volume			Fold change of average normalized volume between groups		Overall fold change of average normalized volume among three groups	
							Normal	CIA	QFGJS	CIA/Normal	QFGJS/CIA	Max/Min^b^	*P* value
1	IgG-2a Ig gamma-2A chain C region	P20760	35677/7.72	11	211	3	6.85	7.45	7.40	3.93	0.90	3.93	3.89*E* − 05
2	Vim vimentin	P31000	53757/5.06	30	282	12	6.34	6.60	6.91	1.82	2.04	3.73	0.011
3	Gstp1 glutathione S-transferase P	P04906	23652/6.89	20	268	3	6.24	6.68	6.55	2.70	0.75	2.70	0.006
4	Apolipoprotein C-III, isoform CRA_b	P06759	7847/4.65	36	163	2	6.03	6.33	5.90	2.00	0.37	2.69	0.011
5	Aldh6a1 methylmalonate-semialdehyde dehydrogenase [acylating], mitochondrial	Q02253	58396/8.44	7	90	3	6.46	6.89	6.56	2.71	0.47	2.71	0.019
6	Ca 3 carbonic anhydrase 3	P14141	29698/6.89	14	109	4	6.46	6.26	6.05	0.64	0.61	2.56	0.026
7	Vdac2 voltage-dependent anion-selective channel protein 2	P81155	32353/7.44	13	104	2	6.45	6.45	6.08	1.00	0.42	2.38	0.045
8	Tpi1 triosephosphate isomerase	P48500	27345/6.89	13	195	2	7.03	6.81	6.71	0.61	0.79	2.10	2.48*E* − 04
9	Ces3 carboxylesterase 3	P16303	62393/6.1	2	88	2	6.52	6.20	6.31	0.48	1.28	2.08	0.035
10	Des desmin	P48675	53481/5.21	7	176	3	7.04	6.73	6.79	0.49	1.15	2.06	0.02
11	RGD1560402 similar to phosphoglycerate kinase 1 (Pgk1)	IPI00372910	43604/6.15	10	97	3	6.63	6.71	6.39	1.20	0.48	2.08	0.034
12	Pgam1 phosphoglycerate mutase 1	P25113	28928/6.67	12	143	3	7.19	7.47	7.43	1.94	0.91	1.94	0.018
13	Anxa2 isoform short of Annexin A2	Q07936-1	38939/7.55	21	172	7	7.01	6.92	6.73	0.80	0.65	1.92	0.015
14	Gpx1 glutathione peroxidase 1	P04041	22472/7.66	16	121	3	6.61	6.88	6.73	1.90	0.71	1.90	0.033
15	Ivd isovaleryl-CoA dehydrogenase, mitochondrial	P12007	46862/8.03	14	112	5	6.86	6.71	6.59	0.70	0.76	1.86	0.002
16	Crp C-reactive protein	P48199	25737/4.89	21	193	4	6.48	6.63	6.75	1.41	1.31	1.85	0.048
17	Capzb F-actin-capping protein subunit beta	Q5XI32	30952/5.69	15	297	5	7.31	7.22	7.04	0.82	0.66	1.83	0.019
18	Idh3a isocitrate dehydrogenase [NAD] subunit alpha, mitochondrial	Q99NA5	40044/6.47	15	199	5	7.01	6.89	6.75	0.77	0.73	1.79	0.027
19	RGD1565368 similar to glyceraldehyde-3-phosphate dehydrogenase	IPI00554039	36045/8.44	12	97	4	7.15	7.01	6.90	0.71	0.78	1.79	0.006
20	Selenbp1 selenium-binding protein 1	Q8VIF7	53069/6.1	16	149	7	7.18	6.99	6.94	0.65	0.90	1.71	0.008
21	Ogn osteoglycin	IPI00362931	34390/5.85	30	284	8	7.64	7.50	7.41	0.72	0.81	1.70	0.007
22	Ldhb L-lactate dehydrogenase B chain	P42123	36874/5.7	11	136	4	7.33	7.20	7.10	0.74	0.81	1.68	0.049
23	Actb actin, cytoplasmic 1	P60711	42052/5.29	13	209	4	7.13	6.99	6.91	0.74	0.83	1.63	0.023
24	Serum albumin	P02770	71244/5.82	9	120	5	6.80	6.73	6.60	0.85	0.73	1.61	0.004
25	S100a4 protein S100-A4	P05942	11997/5.04	52	233	6	7.32	7.53	7.50	1.60	0.95	1.60	0.038
26	Np purine nucleoside phosphorylase	P85973	32566/6.46	12	122	3	7.51	7.64	7.70	1.34	1.15	1.55	0.03
27	Capza2 F-actin-capping protein subunit alpha-2	Q3T1K5	33118/5.57	9	139	2	7.45	7.35	7.27	0.79	0.84	1.52	0.048
28	Mdh1 malate dehydrogenase, cytoplasmic	O88989	36631/6.16	12	73	4	7.27	7.17	7.09	0.80	0.83	1.51	0.024
29	Gsto1 glutathione S-transferase omega-1	Q9Z339	27936/6.25	12	146	3	6.86	6.80	6.70	0.88	0.78	1.46	0.05
30	Apoe apolipoprotein E	P02650	35788/5.23	33	251	11	7.15	7.31	7.24	1.45	0.85	1.45	0.015
31	Aldh2 protein	P11884	53791/5.83	12	396	6	7.35	7.20	7.21	0.71	1.03	1.42	0.045
32	Anxa1 Annexin A1	P07150	39147/6.97	16	316	6	7.38	7.28	7.23	0.79	0.89	1.41	0.033

^a^The differently expressed proteins with fold change of Max/Min less than 1.4 after Progenesis SameSpots software analysis, repetitive proteins analyzed by MALDI-TOF-MS/MS, and unique peptides less than 2 with Mascot software were excluded.

^b^Denote as the ratio of maximum (Max) and minimum (Min) average normalized spot volumes among the normal rats, vehicle-treated CIA, rats and QFGJS-treated CIA rats.

**Table 2:** Classification of the differentially expressed proteins revealed in the knee synovial tissues among the normal and vehicle-treated and QFGJS-treated CIA rats.

Classification	Subclassification	Name of proteins	Functions	Spot no.
Binding	Antigen binding	IgG-2a Ig gamma-2A chain C region	Nucleotide sequence and antibody effector functions	1
	Cytoskeletal protein binding	Anxa2 isoform short of Annexin A2	Calcium-regulated membrane-binding protein	13
	Protein binding	Des desmin	Class-III intermediate filaments	10
		Ogn osteoglycin	Induces bone formation in conjunction with TGF-beta-1 or TGF-beta-2	21
		Actb actin, cytoplasmic 1	Involved in various types of cell motility	23
		Serum albumin	Regulation of the colloidal osmotic pressure of blood	24

Metabolic process	Metabolic process	Gsto1 glutathione S-transferase omega-1	Exhibits glutathione-dependent thiol transferase and dehydroascorbate reductase activities	29
		Gstp1 glutathione S-transferase P	Conjugation of reduced glutathione to hydrophobic electrophiles	3
		Pgam1 phosphoglycerate mutase 1	Catalyze the reaction of EC 5.4.2.4 (synthase) and EC 3.1.3.13 (phosphatase)	12
		Tpi1 triosephosphate isomerase	Belongs to the triosephosphate isomerase family	8
	Lipid metabolic process	Apoe apolipoprotein E	Mediates the binding, internalization, and catabolism of lipoprotein particles	30
	Lipoprotein metabolic process	Apolipoprotein C-III, isoform CRA_b	Inhibits lipoprotein and hepatic lipase and decreases the uptake of lymph chylomicrons	4
	One-carbon metabolic process	Ca 3 carbonic anhydrase 3	Involved in the metabolism of xenobiotics and of natural substrates	6
	Oxidation reduction	Aldh2 protein	Aldehyde dehydrogenase (NAD) activity, identical protein binding	31
		Aldh6a1 methylmalonate-semialdehyde dehydrogenase [acylating], mitochondrial	Plays a role in valine and pyrimidine metabolism	5
		Gpx1 glutathione peroxidase 1	Protects the hemoglobin in erythrocytes	14
		Ivd isovaleryl-CoA dehydrogenase, mitochondrial	It is Oxidoreductase	15
		Idh3a isocitrate dehydrogenase [NAD] subunit alpha, mitochondrial	It is Oxidoreductase	18
		Ldhb L-lactate dehydrogenase B chain	Identical protein, NAD or NADH binding, and L-lactate dehydrogenase activity	22
		Mdh1 malate dehydrogenase, cytoplasmic	Oxidoreductase, NAD or NADH binding, and L-malate dehydrogenase activity	28
		RGD1565368 similar to glyceraldehyde-3-phosphate dehydrogenase	Catalytic activity	19
	Nucleobase, nucleoside, nucleotide, and nucleic acid metabolic process	Np purine nucleoside phosphorylase	Transferase, glycosyltransferase, purine-nucleoside phosphorylase activity	26
	Glycolysis	RGD1560402 similar to phosphoglycerate kinase 1 (Pgk1)	Phosphoglycerate kinase activity	11

Regulation of biological process	Actin filament capping	Capzb F-actin-capping protein subunit beta	Blocking the exchange of subunits	17
		Capza2 F-actin-capping protein subunit alpha-2	Blocking the exchange of subunits	27
	Signal transduction	Anxa1 Annexin A1	Promotes membrane fusion and is involved in exocytosis	32

Cellular process	Protein transport	Selenbp1 Selenium-binding protein 1	Involved in the sensing of reactive xenobiotics	20
	Intermediate filament-based process	Vim vimentin	Plays a role in the stability of the cytoplasmic architecture	2
	Transmembrane transport	Vdac2 Voltage-dependent anion-selective channel protein 2	Forms a channel through the mitochondrial outer membrane that allows diffusion of small hydrophilic molecules	7

Other	Catalytic activity	Ces3 carboxylesterase 3	Involved in the metabolism of xenobiotics and of natural substrates	9
	Acute inflammatory response	Crp C-reactive protein	Displays several functions associated with host defense	16
	Cellular component	S100a4 protein S100-A4	Interacts with PPFIBP1 in a calcium-dependent mode	25

Spot no. denotes the number shown in the first column of Table 1.

The differentially expressed protein spots observed among the normal rats, CIA rats, and QFGJS-treated CIA rats are classified into five classes. 6 proteins are related to binding, 17 proteins are related to metabolic process, 3 proteins are related to biological process, and 3 proteins are related to cellular process and 3 proteins belongs to other class.

## Abbreviations

QFGJS: Qingfu Guanjieshu
RA: Rheumatoid arthritis
MALDI-TOF-MS/MS: Matrix-assisted laser desorption/ionization-time of flight-mass spectrum/mass spectrum
2D: Two-dimensional
(CII): Collagen II
(CIA): Collagen II-induced arthritis.
